# An Interactive Model among Potential Human Risk Factors: 331 Cases of Coal Mine Roof Accidents in China

**DOI:** 10.3390/ijerph15061144

**Published:** 2018-06-01

**Authors:** Ruipeng Tong, Cunli Zhai, Qingli Jia, Chunlin Wu, Yan Liu, Surui Xue

**Affiliations:** 1 School of Resources & Safety Engineering, China University of Mining and Technology (Beijing), Beijing 100083, China; tongruipeng@126.com (R.T.); zhaicunli@163.com (C.Z.); jiaqingli@163.com (Q.J.); 2 School of Economics and Management, Beihang University, Beijing 100191, China; 3 Beijing Key Laboratory of Emergency Support Simulation Technologies for City Operations, Beihang University, Beijing 100191, China; 4 Faculty of Civil Engineering and Geosciences, Delft University of Technology, Stevinweg 1, 2628 CN Delft, The Netherlands; y.liu-9@tudelft.nl; 5 School of Safety Engineering, China University of Labor Relations, Beijing 100048, China; xuesurui@126.com

**Keywords:** human risk, potential factor, interactive model, knowledge level, information integrity, communication degree

## Abstract

In order to explore optimal strategies for managing potential human risk factors, this paper developed an interactive model among potential human risk factors based on the development processes of accidents. This model was divided into four stages, i.e., risk latency stage, risk accumulation stage, risk explosion stage and risk residue stage. Based on this model, this paper analyzed risk management procedures and relevant personal’s responsibility in each stage, and then probed into the interactive mechanism among human risk factors in three aspects, i.e., knowledge, information and communication. The validity and feasibility of the model was validated by analyzing a coal mine roof accident in China. In addition, the contribution of different functional levels’ personnel in risk evolution was discussed. It showed that this model can effectively reveal the interactive mechanism of potential human risk factors, and can thus give significant insights into the development of risk management theories and practices. It also proves that the contribution of different functional levels’ personnel in the model is different. This can further help practitioners design enhanced Behavioral-Based Safety (BBS) intervention approaches which can have a more sustainable and persistent impact on corporate personnel’s safety behavior. Specific recommendations and suggestions are provided fundamentally for future BBS practices in the coal mine industry.

## 1. Introduction

In the course of risk management research development, the definition of risk has been changing continuously, including uncertainty [1], event consequence [2], risk probability [3], expected loss [4], etc. At present, the risk definition, which centered on the occurrence probability of risk events, has become the guideline of risk research. For example, risk was the uncertainty of the results, actions and events [5]. The risk was also defined as the occurrence probability of unexpected events by Campbell [6].

With the deepening of risk management research in many disciplines, researchers have already systematically combed and analyzed risk perspective, risk description, risk concept scope, risk research objectives [7,8], etc. They have also classified the risk research methods into qualitative methods, quantitative methods and mixed methods [9]. Quantitative risk analysis is dominated by risk probability assessments. Given the limitations of qualitative risk analysis methods and the great gaps between the method and its application [9], quantitative risk analysis gradually overwhelms the pure qualitative analysis and becomes one of the major approaches to measuring risks and in turn instructing risk management [10].

Risk events are characterized by uncertainty and unpredictability [11]. For different types of industries, inducing factors of risks are closely linked to the corresponding industrial background, and pre-accident omens or various antecedents are likely to be hidden in the production process. Therefore, it’s essential to identify risk factors comprehensively and effectively in risk management [10]. However, in the risk analysis process, not all risk factors can be measured or quantified, and the effectiveness and completeness of risk assessment results depend on how well the risk model matches the real situations [12]. In the previous studies, the quantitative risk model achieves risk scene simulation or accident reconstruction by artificially simplifying practical problems and subjectively setting various assumptions [13,14]. This makes risk assessment results obtained by these models deviate greatly from the actual situation [15]. Small probability events, called black swan events, are even generally ignored [16]. Nevertheless, in the real risk management process, the corporate personnel are more inclined to depend on the risk probability value obtained by the above models [17]. As a matter of fact, errors and flaws still exist within the process of risk quantification and risk management [18].

More and more researchers also admit that the risk depends on the subjective judgment of analysts, that is, the knowledge of risk analysts [19]. In risk management, due to personal’s lack of cognition of risk information and their insufficient information exchange in their own enterprises, they fail to take effective actions timely to control the risk in the pre-accident omen period, and thus eventually lead to accidents. This phenomenon is particularly prominent in the coal industry of China, where coal mining remains one of the high-risk industries [20]. With strict supervision of the coal industry by the national government, the fatality and injury rates have been reduced to some extent. Nonetheless, thousands of workers are still killed in coal mining incidents every year. The number of accidents per one million population in China was 37 times higher than that in the U.S., in the period from 2001 to 2008 [21]. Wei [22] stated that the fatality rate in China was 88 times higher than in the U.S., by comparing the mortality rates per million tons in the two countries, in the period from 2000 to 2009.

Currently, risk management in coal mining in China mainly includes risk identification, risk assessment and risk pre-control [23]. In the process of risk identification, because of the limited knowledge of personnel, the identification of risk is also associated with blind areas, namely the unrecognized risks that may have an important impact on safety management. In the process of risk assessment, the quality of risk assessment is poor because of insufficient information collected, and in turn results in the failure of really understanding the risk status of the corporation, let alone taking risk controlling measures. In the process of risk pre-control, there are huge differences in the understanding of risk between the practitioners, so they do not handle all risks in appropriate manners.

Moreover, accident investigation results indicated that the unsafe behavior of workers is still the dominant direct cause of the coal mining accidents [20,24,25,26,27]. Behavior-Based Safety (BBS) is an approach to control or eliminate human unsafe behaviors by intervening and modifying human unsafe behaviors [28]. The BBS approach is drawing increase attention in the area of occupational safety. Generally, the processes implemented by BBS is to: (a) define key behaviors; (b) establishing BBS observation teams; (c) observing and collecting data on behaviors; (d) analyzing the causes of unsafe behavior; (e) feeding back unsafe behavior information; (f) correcting unsafe behavior; (g) verifying the effectiveness, which ultimately reduce the accident rate. These days, BBS has been applied successfully by many researchers to clinical medicine [29], manufacturing [30], petro-chemistry and mining [31]. These successful implementations proved the effectiveness of BBS on modifying personals’ unsafe or at-risk behaviors. It also was considered an effective way to further prevent accidents. Krause et al. investigated the five years of injury data from 73 corporations who implemented BBS and their results revealed a significant reduced accident across groups after the BBS approach implementation [32].

However, it was found that the BBS approach is currently facing a key difficulty in achieving continuous intervention effects. Although better behavior performance can be achieved during the implementation of the intervention, behavior performance deteriorates significantly after the removal of the intervention. The reason for the above questions is that the BBS approach focuses on the personal’s goal setting and performance feedback, ignoring other factors that influence the effectiveness of the intervention. That is, it has not solved the fundamental problems leading to the unsafe behavior. Once the intervention is removed, the potential factors that caused the unsafe behavior to take place, once again take the dominant position [33]. Zhang et al. indicated that more consideration should be given to potential factors affecting unsafe behavior, thereby improving the effectiveness of behavioral interventions [34]. Potential human risk factors are one of these factors. It exists in the whole production process and is accumulate and superpose. Therefore, it is of great significance to analyze the potential human risk factors related workers unsafe behavior.

Over ten years of statistical data (State Administration of Coal Mine Safety (SACMS)) show that in China, the proportion of coal mine roof accidents in the total number of occupational accidents was 51.2%, and the number of deaths caused by coal mine roof accidents accounted for 36.5% of the total deaths [35]. Therefore, this study took coal mine roof accidents as the research object and summarized 331 cases of coal mine roof accidents occurring in China from 1983 to 2014 [35]. Simultaneously, unsafe behaviors and unsafe actions in these cases were analyzed, and potential risk factors related to human behavior were further explored. After that, the interactive model among potential human risk factors was built based on the results of above analysis in this paper to discover the interactive mechanisms among potential human risk factors, and then the rationality of the model is validated by analyzing a coal mine roof accident. Moreover, this paper also provides an in-depth analysis in term of the interactive impact of the potential human risk factors among different functional levels’ personnel by analyzing the correlation among its behaviors. It is expected that this result can give more significant insights into the development of risk management theories and practices, that specific recommendations and suggestions are provided for future BBS practices in the coal mine industry, and that can further help practitioners design enhanced BBS intervention approaches which can have a more sustainable and persistent impact on worker safety behavior.

## 2. Methods

### 2.1. Emergence of Potential Human Risk Factors

In the development of risk research, there are multiple definitions of risk because the different ways of understanding this concept. Some risk definitions are based on probabilities, opportunities or expected values, some are based on undesirable events or dangers, and others are based on uncertainty [36]. Therefore, by reviewing the risk-related literature, the risk definition was divided into nine categories, which can cover the risk connotation to a large extent [13]. It was summarized that risk-related factors are uncertain, undesirable events and the probabilities of their occurrence. That is to say, it is uncertain to turn the risk into an unexpected event, and the consequences of the undesirable event are also uncertain. Uncertainty is represented by probability value during risk analysis.

In the actual risk management process, practitioners identify the risks that exist in the corporation and use risk assessments to obtain the probability that the risks will be converted into unexpected events. If the probability value is greater than the expected value, then the risk is not accepted and needs to be managed and controlled. If the probability value is smaller than the expected value, the risk can be been accepted. However, the risk cognition level of the relevant personal has a significant impact on the accuracy of the assessment results and the effectiveness of the controlling measures [11]. The risks in the corporation cannot be fully identified since the limitation of practitioners’ knowledge level [25]. Veland et al. [11] found through a large number of investigations that there is a big difference between risk cognitions of different people, and that some have a higher level of risk cognition, but others, due to poor communication or lack of information leading to the emergence of knowledge hollow, have a lower level of risk cognition. Thus, the potential human risk has generally attracted attention.

Vinodkumar et al. [37] found the importance of practitioners’ safety knowledge level and then proposed that safety management practices such as safety training, safety communication and safety rules and procedure execution must be designed to improve practitioners’ safety knowledge. Dahl [38] also pointed out that the reason for unintentional violations is that practitioners’ knowledge of rules and procedures is poor. Therefore, Aven [39] took the impact of personal’s risk cognition level into consideration in risk assessment and risk management. He then proposed R = (A, C, Q, K), where A means risk events, C means risk assessment results, Q means risk values, and K means risk assessment related knowledge [37]. Moreover, Allanson [40] indicated that sufficient information should be obtained during production to prevent an accident occurring after investigating various accidents. Kines et al. [41] found that foremen to include safety in their daily verbal exchanges with workers had a significantly positive effect on their behavioral safety performance in construction site. Communication plays an important role in security work. Therefore, Zhang et al. [34] recommend that when observers find someone performing unsafely, they should communicate with the worker in good faith, point out the unsafe behavior and suggest ways to work safely. Li et al. [42] found that efficient risk communication is an important way to reduce the vulnerability of individuals when facing an emergency risk. Even Jiao et al. [43] has used an interactive customized portal as a risk communication tool to enable residents to understand the potential risks of nearby industries. 

In summary, the knowledge relates to safety production, involving safety techniques, safety rules and procedures, risks associated with production, risk disposal measures, and other safety-related content. Information refers to the real performance of man-machine-environments-management in the safety production process, such as objective job site conditions and personal behavior. Communication is an effective and simple way of communicating with staff of the same or different levels. These three concepts are related to each other. A man has sufficient knowledge to help them discover and fully collect information that threatens safety production, and make corresponding decisions. Simultaneously, according to the information that has been obtained, you can continuously improve your knowledge. Among them, communication is a channel for collecting and processing information and obtaining knowledge. For example, managers communicate with employees at the frontline level to obtain the real status of the work site and make the information obtained more accurately. Managers pass necessary safety skills to employees at the grass-roots level through the communication to enhance the knowledge level of the employees. At the same time, when employees discover information about other employee’s unsafe behavior based on their own knowledge, they can communicate with them to achieve the purpose of correcting their unsafe behavior. Consequently, the knowledge, communication and information related to personal are considered as potential human risk factors, which have a significant impact on risk assessment and risk management. The following content is further studied.

### 2.2. Analysis of Potential Human Risk Factors in Empirical Data

Through the analysis on a large number of empirical data collected from coal mine roof accidents [24,25,26], the working mistake or violation behavior performed by workers or relevant managers are the direct causes of accidents. Further, potential risk factors leading workers or managers to producing a series of unsafe behaviors exist in the whole operation process. Various accidents eventually occurred after these factors were gradually accumulated and superposed in different periods. A total of 331 coal mine roof accidents were analyzed from 1983 and 2014 in China. The details of those are shown in Table 1.

Table 1 show that there were two main types of unsafe behavior, i.e., workers’ violation operations and managers’ violation commands. Namely, the low safety quality of workers and managers’ mistakes in supervision and command work are the common causes of coal mine roof accidents [11,44]. In the complex working environment of coal mines, workers face complicated work procedures and heavy workloads. Therefore, potential human risk factors occupy an important position in the accident, and the specific analysis is as follows.

#### 2.2.1. Violation Operations of Workers

The most frequent unsafe actions of operators were use of improper supporting structures (272 accidents) and careless inspections during safety inspection (109 accidents), due to their lack of information about the operating environment. The following ones are listed as: handling roof-fall accidents illegally (63 accidents), ignoring supervision (59 accidents), illegal blasting (25 accidents) and so on, because of the limitation of their own safety knowledge. Then the general unsafe actions were cross operation (17 accidents), standing in a wrong place (15 accidents), tunneling in a wrong way (3 accidents) and so on, as a result of the poor communication about work content.

#### 2.2.2. Violation Commands of Managers

In an unfamiliar operating environment situation, the most frequent unsafe actions caused by managers were issuing illegal work commands (90 accidents), failure to supervise work in a timely way (69 accidents), failure to formulate complete safety measures (51 accidents) etc. Moreover, some of the behavior reflected the safety quality of managers should be improved, such as not organizing an evacuation when faced with a clear sign of roof collapse (20 accidents), being equipped with insufficient staff (8 accidents), keeping escape exits closed (2 accidents).

### 2.3. Research on Potential Human Risk Factors Model

**Figure 1:**
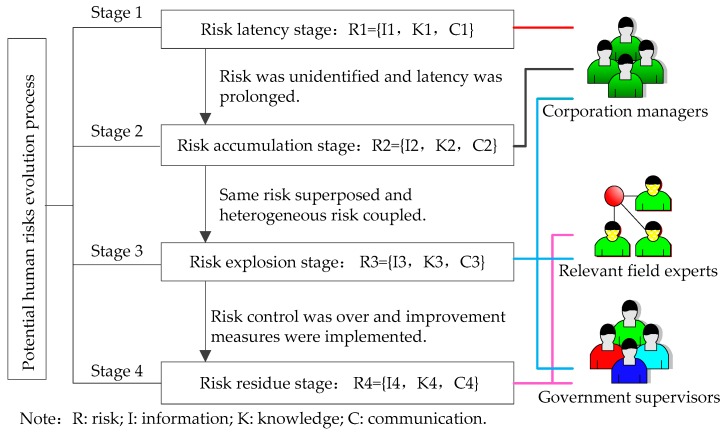
Interactive model among potential human risk factors.

Through the in-depth analysis of 331 coal mine roof accidents, the interaction among potential human risk factors leads to the occurrence of unsafe behavior (workers’ violation operations, managers’ violation commands). These potential human risk factors include knowledge level, completeness of information and degree of communication. The potential human risk factors need to undergo a certain evolution [45], and then induce a coal mine roof accident. The specific evolution model is shown in Figure 1.

#### 2.3.1. Risk Latency Stage

The coal industry is considered to be the industry with the highest risk in the world [46,47]. In the process of coal mine production, the operating environment is complex and the operating conditions can change at any time [48]. Therefore, a series of potential risk factors gradually affect each operation link, such as the process parameters deviating from the normal value [49], abnormal equipment operation and the operating environment which does not meet the safety standards [50,51]. However, workers were lack of necessary risk knowledge or safety knowledge, and they couldn’t identify risk information effectively. Further, they failed to communicate with the superior managers. In consequence, the operation group members failed to adjust the work program or take relevant emergency measures timely. Eventually, potential human risk factors exist and are not found. Potential risk of this period was defined as R1. Related interactive factors were defined as knowledge (K1), information (I1) and communication (C1).

#### 2.3.2. Risk Accumulation Stage

In the process of risk latency, without taking any risk mitigation measures, potential risks gradually accumulate with time and show a dynamic change, such as process parameters getting close to the safety threshold [49], safety devices gradually losing their original function [52], increased risk categories in the operating environment [53]. Because workers had little knowledge of the safety rules of operation, these signs of risk couldn’t be found in a timely fashion. They also could hardly take timely and effective measures for replying to these signs, because they had little knowledge about the safe operation technology. In addition, junior managers are often focused on production, completely ignoring potential risks or risk signs in the operational chain. Even these signs of risk were considered as a normal phenomenon in the production process and didn’t trigger other bad consequences [54]. Therefore, they have neither controlled the risk nor reported them to their superiors. As a result of that, the same risk continued to be superposed, the heterogeneous risk sustained to be coupled. Potential human risk of this period was defined as R2. Related interactive factors were defined as knowledge (K2), information (I2) and communication (C2).

#### 2.3.3. Risk Explosion Stage

In the process of upgrading risk accumulation, the workers missed the best opportunity to manage and control the risk, leading to continued accumulation of potential human risks. Then risk fusion occurred at some timepoint, and potential risks eventually evolved into a risk event. At this point, the enterprise started emergency plans to prevent the risk event from expanding [55]. As the exchange of information was ineffective in the process of collaborative linkage among different departments, and the relevant personnel in charge had a unilateral cognition level of the risk event, emergency plans didn’t achieve the same effect as expected and then the risk event evolved into an accident. Later, government departments and relevant experts become involved in the emergency rescue. In order to make the rescue operation in an orderly and efficient manner, it is necessary to comprehensively assess the accident situation which includes the environment characteristics of the accident site [56], the characteristics of the production operation of the accident enterprise and the evacuation of the accident site personnel [57], etc. However, decision layers and rescuers failed to grasp or predict the trend of the accident effectively, and even ignored some possible accident situations, as a result of their lack of necessary risk knowledge or information. Those are important potential factors leading to accident expansion or secondary accident. Potential risk of this period was defined as R3. Related interactive factors were defined as knowledge (K3), information (I3) and communication (C3).

#### 2.3.4. Risk Residue Stage

After accident rescue, experts in relevant fields have investigated and collected evidence on the accident site. This accident report provided an important theoretical basis and technical support for finding production operations loopholes, analyzing accident development processes, investigating the effect of accident rescue [58], etc. Based on these, after completing the accident handling, there are a lot of works to be done during the subsequent production. First, risk assessment is to be completed for the operating conditions of industrial park [59], factory area or workshop [60,61]. Second, determining whether the risk of production conditions is acceptable. Third, referred to similar industry management specification or relevant industry demonstration cases, enterprises should take a series of risk mitigation measures. These measures involve normative documents or practical improvement plans of human, machine, environment, management and other aspects. However, in the course of implementing the control measures, it is worth noting that the interaction among governmental supervisors, enterprise managers and workers is an important factor affecting the effect of risk control. Potential human risk of this period was defined as R4. Related interactive factors were defined as knowledge (K4), information (I4) and communication (C4).

The above is the interactive influence of potential human risk factors in the process of the accident. Among them, the model mainly involves human factors. Practitioners are the most direct links to risk in corporate management. In addition, there are differences in the effected scope and intensity of behavior performed by practitioners in different positions. Therefore, it is necessary to deeply elaborate the interactive impact of the potential human risk factors in different functional levels’ personnel.

The Pearson correlation coefficient method is a common linear correlation coefficient. R is generally used to indicate the degree of correlation between two variables. Its values are within (−1, 1), among which *r* = 1 means a completely positive correlation, *r* = −1 means a completely negative correlation, and *r* = 0 means being not correlated at all. Thus, to ascertain the degrees of correlations among unsafe behavior produced by different functional levels’ personnel, the method of correlation analysis was used by the SPSS software to analyze the 32-year coal mine roof accidents data. The specific research results will be shown in Section 4.

## 3. Model-Based Case Study

### 3.1. Case Introduction

#### 3.1.1. Accident Profile

A major roof accident (hereafter called “4-24” major roof accident) occurred at about 4:40 p.m. on 24 April 2008 in the Yuchi Coal Industry Co., Ltd. facility in Jincheng, China, killing 10 miners, injuring 2 and causing direct losses of 6.569 million yuan,. According to the investigation, the accident was located at the intersection of a haulage gate and an old roadway on the 3111 face. This major coal mine roof accident was identified as a liability accident by the accident investigation team.

#### 3.1.2. Accident Process

The coal mine is a legal mine with high productivity, adopting roof caving technology of long-wall retreating cantilever support, applying the full collapse method to roof management and using side driving and side support operations and three-shift cyclic working in the tunneling process. Production faces include a coal face and two heading faces, namely, 3410 for the coal face, 3111 return air gate and 3111 haulage gate for the heading face which is supported by wooden shedding. The end of the 3111 face is the naked old roadway excavated in the 1990s. When the 3111 return air gate was completed on 16 April 2008 and haulage gate was completed in the night on 23 April 2008, they had run-through the naked old roadway and 3111 haulage gate. Namely, a five-roadway intersection was formed. However, after run-through working, workers were outside the rules of operation and operation technique to support the naked old roadway. At 14:30 p.m., after the pre-working meeting, the captain of tunneling team and squad leader led 19 people (safety officer included) to reach the intersection and began work, but none of them had received safety training. Then the captain of tunneling team left. After blasting, the support collapsed. In conditions that neither checked the operating environment nor repaired the support in time, the squad leader ordered operators into the naked old roadway to clean coal. Workers were working under the unsupported roof at the time of the roof collapse.

### 3.2. STEP-Based Accident Description

Sequential Timed Events Plotting (STEP) is a multi-linear event sequence model and method. It stores accident-related events through worksheets and explains the incident [62]. The STEP worksheet is a simple matrix. In the worksheet, the leftmost column has the name of the actors, the top row is a time series axis, and the squares in the diagram represent the event which is defined as an actor or action.

**Figure 2:**
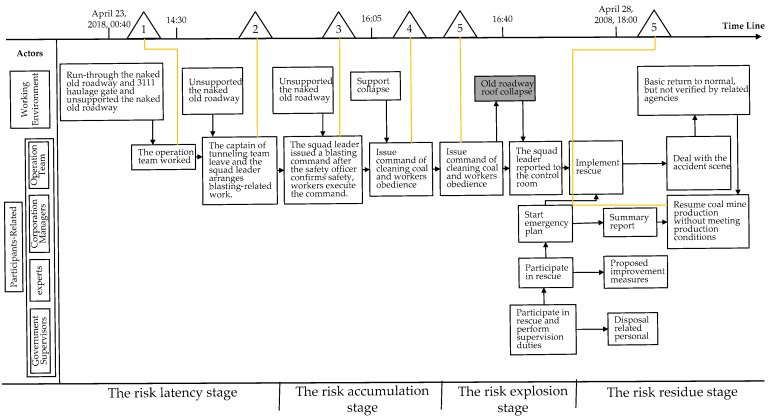
The development of the “4-24” major roof accident.

Based on the investigation result of the “4-24” major roof accident, it can be found that the actors involved in the accident can be mainly divided into working environment and participant-related. Among them, the working environment is the environmental conditions in the run-through of the haulage gate and naked old roadway in the 3111 heading face, and the practitioner-related ones include the members of the operation team, corporate managers, relevant field experts and government supervisors. For these actors, the related events/behaviors were categorized in chronological order, and the logical relationship between them was analyzed. The development of the “4-24” major roof accident was described by the STEP method as shown in Figure 2.

The development of the accident was clearly demonstrated by applying the STEP method. By judging whether each event/behavior is in a safe state, the events/behaviors in an unsafe state are marked with triangles in Figure 2 as an entry point for the next phase of a bow-tie analysis.

### 3.3. Accident Risk Analysis

The accident investigation shows the background information of this coal mine, the process and cause of the roof collapse, the emergency rescue process during the accident, the disposal plan of the responsible persons, and preventive measures for preventing the occurrence of similar accidents. The causes of the accident in this investigation were analyzed from the direct and indirect causes. It was considered the direct cause was that the naked old roadway was not supported and workers worked under unsupported roofs. Simultaneously, it was considered the indirect reason was that corporate leaders did not pay attention to safety, and that the relevant government and corporation personnel failed to supervise work in a timely way. However, the report also has some deficiencies. For instance, the indirect causes are not specific, and there is also little analysis of the human factors that are the most closely related factors to unsafe behavior.

Therefore, based on the investigation result of “4-24” major roof accident and Figure 2, accident reasons were analyzed by bow-tie software. The coal mine roof accident was recognized as a top event. The main threat include that naked old roadways weren’t supported, that related workers didn’t accept safety training, that workers worked under a roof lacking support, etc. Active barrier measures were ignored by relevant personal. As for the result, a coal mine roof accident occurred. Based on the interactive model, the bow-tie software analysis results were appropriately adjusted, as shown in Table 2.

Based on the above analysis results, preventive measures and suggestions to improve the effectiveness of risk management can be proposed from three aspects, i.e., improving the knowledge level of practitioners, enhancing the integrity of information collection, and encouraging full communication:

- There are many ways to improve the knowledge level of corporate practitioners and government supervisors. Among them, safety education and training are the most common methods. The content of training should be rich and the language should be easy to understand.
- Make full use of the functions of mobile phones, newspapers, instruction manuals and other media to transmit safety knowledge to corporate practitioners and government supervisors.
- Formulate a strict knowledge level assessment system, and those who fail to pass the assessment should not be allowed to work.
- The government should regularly conduct a comprehensive inspection of all types of production infrastructure coal mines under its jurisdiction to understand the coal mine risk information. Corporate practitioners are required to monitor operating the environment information in real time.
- Formulate corresponding documents to ensure the smooth flow of communication channels among corporate practitioners.

## 4. Impact of Different Functional Personnel on the Interactive Mechanism

**Figure 3:**
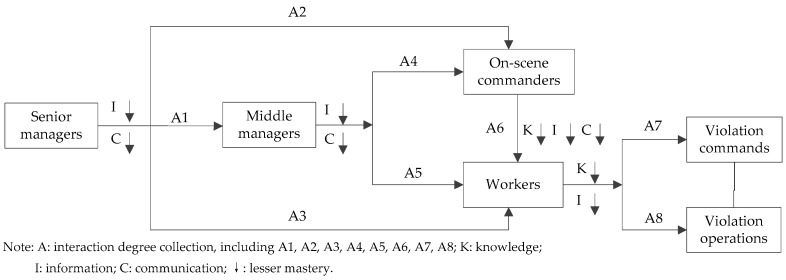
The interaction of potential human risk factors on the different functional levels’ personnel.

Potential human risk factors which are generated by personnel of different functional levels have a significant contributing to personal behavior. In the corporation, the affected scope and intensity of unsafe behavior performed by different functions are different. The level of knowledge, information, and communication demonstrated by personnel of different functional levels have different effects on the evolution of potential human risk factors in the model. To further analyze the role of personnel in the model, relevant corporation practitioners were divided into workers, on-scene leaders, middle managers and senior managers. The interactions of potential human risk factors on them are shown in Figure 3.

As is shown in Figure 3, potential human risk factors act on senior managers, middle managers, on-scene leaders and workers, and these factors play an important role in the transmission of practitioners’ behavior. Therefore, the correlation of unsafe behaviors was analyzed to discuss the role of potential human risk factors. Three hundred and thirty one (331) coal mine accidents were collated (Table 1) on the basis of unsafe behavior’ producers, and then analyzed by applying the Statistical Package for Social Sciences (SPSS) software (International Business Machines Corp., New York, NY, USA).

The correlation of unsafe behavior produced by different functional levels’ personnel in coal mine was quantified by interaction degree collection, Pearson correlation coefficient and its test of significance (*p* value). The correlation analysis results of unsafe behaviors are shown in Table 3. The analytical results show that the behavior correlation coefficients are all highly correlated (*r* > 0.8), and that the eight correlation coefficients are all significant at the 0.01 level (2-tailed). The specific analysis is as follows.

### 4.1. Level One

Senior managers play an overview role in corporations and they are the decision makers who formulate the major safety decisions and development policies of the enterprises. However, their cognition of industry situation and mastery degree of enterprise development is an important information support in the process of taking important decisions [63]. Therefore, during this process, the lack of information would lead to decision-making mistakes. Meanwhile, it could affect the work arrangements made by middle managers (*r* = 0.941, *p* = 0.000). Furthermore, with the guidance of one-sided information, there were large limitations and constraints in communicating content of middle managers and senior managers. That increased spatial dimensions and time scales of potential risk [64]. In addition, the overall consciousness of senior managers not only affected the enterprise development concepts, gradually impact on the on-scene leader (*r* = 0.882, *p* = 0.002) and operators (*r* = 0.961, *p* = 0.002). In this case, the risk exists in the corporation’s safety decisions, and then extended to the entire production.

### 4.2. Level Two

Successful operation of the corporation depends on the correct decision-making and effective implementation. Table 3 shows that the behavior of senior managers affects the behavior of middle managers (*r* = 0.941, *p* = 0.000), the behavior of middle managers also affects the behavior of on-scene leaders (*r* = 0.967, *p* = 0.000) and workers (*r* = 0.904, *p* = 0.000). Therefore, middle managers are the link between senior managers and grassroots personnel, who are the performers of the enterprise strategy decisions. At the same time, their suggestion is an important basis for senior managers to make decisions. If the middle managers lack the necessary knowledge of production safety, are not familiar with the production environment information, lack communication with the upper and lower levels or communication is not complete, then they have no ability to find the risks lurking in decision-making or cannot fully understand the meaning of decision-making, they also failed to give the correct commands to the on-scene leaders or workers, so that impacted workers’ operating procedures and progress (*r* = 0.904, *p* = 0.000).

### 4.3. Level Three

At the operation site, operation progress, operation mode and the specific behavior of workers (*r* = 0.925, *p* = 0.000) depend on the on-scene leader. However, judging from the data of available coal mine roof accidents [24], workers are at a low level of cultural quality and operational level [65]. Then they fail to fully understand the macro-scheduling commands and operational details at the operating site. The on-scene leader failed to identify potential human risk factors in the operational environment, operation process and workers. Meanwhile, they were more prone to violate commands or forced workers to perform the relevant commands (*r* = 0.950, *p* = 0.004) in pursuit of production efficiency and operating progress.

### 4.4. Level Four

Workers’ behaviors were affected by such factors as senior managers’ safety policies (*r* = 0.961, *p* = 0.000), middle managers’ requirements for measures implementation (*r* = 0.904, *p* = 0.000) and on-site leaders’ supervision (*r* = 0.925, *p* = 0.000), etc. Workers with different functions had a greater difference in knowledge and information. Meanwhile, the exchange of all levels of managers was limited to implementing system measures, without being associated with the actual operating situation and the real development of the enterprise [44]. This caused the operating instructions to deviate from the real situation, so that workers failed to identify the risk factors in the operations in a timely way. In addition, safety education and training were not well implemented for the workers, which led to their safety knowledge and skills not having been substantially improved. They even had little ability to deal with any abnormal operation situation in a timely and effective manner. Besides, heavy work tasks made workers be burdened with a huge mental load and physical load, which caused them to be overfatigued, suffer mental confusion or violate original safety working procedures (*r* = 0.901, *p* = 0.000) for improving work efficiency. That is why operation violations become the cause of many accidents.

## 5. Discussion and Implications

### 5.1. Validity and Reliability of the Research Model

In order to improve the effectiveness of risk management from the perspective of behavior, it is necessary to regulate practitioners’ behavior so that they show safe behavior at work. This article aims to build and validate an interactive model among potential human risk factors. This model analyzed the impact of potential human risk factors on human unsafe behavior in the process of accidents, and further analyzed the interactive impact of the potential human risk factors in different functional levels’ personnel.

Lin et al. [66] built an integrative model of safety management based on social cognitive theory and the total safety culture triadic framework. They revealed a causal relationship between a hazardous environment, safety climate, and individual safety behaviors. Bosak et al. [67] analyzed the impact of safety climate (management commitment to safety, priority of safety, and pressure for production) on practitioners’ risk behaviors. They found that practitioners’ risk behavior is negatively related to management commitment to safety and priority of safety, and that behavior is positively related to pressure for production.

Through the analysis of the above research results, it can be seen that the dangerous environment is the work-related information that practitioners must understand, and that management commitment to safety and priority of safety are the decisions made by management through its own knowledge, combined with information gathered at the work site and communicated with other practitioners. High-intensity pressure for production is bad information for practitioners, so according to this information, timely measures are taken to reduce production pressure and ensure practitioners behavior safety.

In addition, Babette et al. [68] proposed a new, multifaceted intervention strategy for improving safety behaviors, including senior managers, supervisors and employees. These intervention strategies are mainly focused on changing supervisory communication with workers, which is consistent with the emphasis on the direct role of direct manager in the security climate [69]. Other scholars also emphasized the influence of other security agents, such as employees and senior managers [70,71]. Brondino et al. [72] also examined the multifaceted nature of the concept of safety climate and demonstrated that it involves multiple levels of organization, including senior management and workers. Therefore, the above interventions highlight the influence of different functional levels’ personnel and the important role of communication and interaction. This is consistent with the point of view of the model derived from this article. It can be concluded that one of the objectives of the study of the interaction of knowledge, information and communication in this article is to achieve a good working environment.

### 5.2. Major Findings

The research in this paper was designed to explore interactive mechanisms among potential human risk factors, and thus give insights into influencing factors of unsafe behavior and the behavioral influence relation among different functional levels. In light of the findings from this paper, knowledge, communication and information serve as the potential human risk factors. Interactive model among potential human risk factors explains the interactive mechanism among knowledge, communication and information. On the whole, the feasibility of the model was fairly validated. Major findings of this paper are summarized and discussed, as follows.

Knowledge, communication and information are the major expression of potential human risk factors in coal mine, and exist throughout the production process. The lack of knowledge led to the fact that the relevant personnel failed to identify effective information such as risk factors in the operating environment. The relevant personnel performed unsafe behavior such as violating operations, violating commands, etc., so that they missed the best time to manage and control the risk. In addition, the lack of knowledge led to the low level of personnel’s risk cognition, and thus affected effective communication. Hence, the grassroots situation is delayed feedback to the senior management, leading to management decision-making that deviated from the real production situation. Potential human risk factors has experienced the risk latency stage, risk accumulation stage, risk explosion stage and risk residue stage, and exist in the whole process of coal mine accidents.

The closer the function level is, the more significant impact of the personnel’s behavior. The effect of senior managers on middle managers (*r* = 0.941, *p* = 0.000) is greater than that on on-scene leaders (*r* = 0.882, *p* = 0.002) and workers (*r* = 0.961, *p* = 0.002). The effect of middle managers on on-scene leaders (*r* = 0.967, *p* = 0.000) is greater than that on workers (*r* = 0.904, *p* = 0.000). The commands given by on-scene leader directly determine the behavior of the workers (*r* = 0.925, *p* = 0.000). The behavior of the senior managers (*r* = 0.961, *p* = 0.002) have the greatest influence on the workers’ behavior, followed by the influence of the on-scene leaders (*r* = 0.925, *p* = 0.000), and the middle managers (*r* = 0.904, *p* = 0.000) have the least influence. Further, knowledge deficiency, information shortage and inefficient communication of personnel represent the latent risk in the corporation. More specifically, senior managers’ performance is the beginning of the potential human risk evolution. Middle managers’ behavior plays a vital catalytic role. The behavior of on-scene leaders and workers translates the potential risk directly into an accident. After that, all levels of personnel will to find the reasons and for the preparation for resume production.

### 5.3. Theoretical Implications

Findings of the research in this paper are of great significance to the analysis of personnel unsafe behavior. Previous studies have explained that knowledge, communication and information have an important impact on risk cognition or personnel behavior. Personnel safety knowledge level is different, their risk cognition is different [73]. The prerequisite for good risk understanding is sufficient professional knowledge [74]. When workers discover information such as risk and unsafe behavior on site, it can be fed back to other workers through communication and helps them get out of danger instantly [75]. However, although the importance of unsafe behavior to accidents has been well-established, the interactive mechanism by which the potential human factor affects the safe behavior is less clear. How the potential human factors i.e., knowledge, communication and information, affect the personnel behavior, which in turn evolved into an accident? This paper made a substantial contribution to answering the question.

Furthermore, findings of the research in this paper have significant implications for human factors research in coal mine accidents. Many previous studies [46,76] have analyzed the cause of coal mine accidents in term of unsafe human behavior. However, safety is not only affected by the workers alone, but also by decisions at all levels made by managers, safety officers [77], etc. Senior managers are the decision maker to develop the major safety decisions and development policies of the enterprises. If the decision made by senior managers is inappropriate, it will develop toward unsafe for the entire production environment and personnel behavior. The middle manager is the hub that associates senior managers and grassroots personnel. If the behavior of middle managers is at risk, it will directly affect the quality of major decision-making. On-scene leaders and workers are exposed directly to dangerous workplaces. If the risk is not found or the measures taken are invalid, the risk will not be controlled or eliminated in the previous levels. In the next levels, the unsafe behavior of workers and on-scene leaders is the direct factor that the potential human risk is transformed into an accident. Therefore, decision-making is unreasonable made by senior managers in their knowledge deficiency, inefficient communication and information shortage. That is the deep cause of the evolution of potential human risk factors into an accident. The unsafe behavior of middle managers is the indirect reason. The unsafe behavior performed by on-scene leaders and workers is the direct reason of the accident.

### 5.4. Practical Implications

The research in this paper focuses on the potential human risk factors that can impact personnel’s safety behavior, thus evolve into an accident. Therefore, it is of great practical significance to prevent accidents. Corporate personnel can design innovative and BBS intervention schemes based on potential human risk factors. This paper shows that knowledge deficiency, inefficient communication and information shortage are the deep cause of accidents. This was also verified by previous studies. After experiencing the accident latency phase, accident expansion phase and emergency rescue, more knowledge defects of workers were exposed in the accident risk disposal [44], i.e., the hazard factors couldn’t be identified effectively in operating environment, thus further increasing the risk of accidents in the latency stage. When accident risk expanded gradually and appeared a series of signs, workers failed to take effective control measures in time. As a result, the potential risk evolved into an accident. In the case of insufficient information, grassroots managers or workers mistook for higher safe of operating environment, which led to many adventure operations such as working under empty-supporting roof, roof repair delay [24], etc. Because the managers in township coal mines or state-owned local coal mines had little risk knowledge or safety knowledge, they had little ability to control the potential risk in their own coal mines from a global perspective [78]. Safety education and training for personnel means improving the level of their knowledge and improving their skills. It is critical to standardize personnel behavior [79]. It is of great significance to prevent accidents that take risk control measures in terms of knowledge, information and communication. The specific measures are as follows:

*Safety education and training specialization.* Because the different positions require different job quality, the training object is divided into four levels, i.e., senior management, middle managers, on-scene leaders and workers. In the process of optimizing the knowledge system of coal mine personnel, the safety education and training content of the personnel in different functional levels should match the job requirements. The content of the training of senior managers needs to focus on the dialectical relationship between laws and regulations and personal responsibility, the game relationship between safety investment and economic benefit. The training content of middle managers should focus on the game relationship between risk factors and coal mine accidents, the matching degree of enterprise system and real operation situation, the rationality of personnel distribution. On-scene leaders and workers should receive training in operation procedures, safety practices and other aspects.

*Enhance communication between personnel.* The communication between different functional levels was related to the coordination between the organizational departments. Enterprises according to their actual situation can improve the organizational structure, and clarify the responsibilities, rights and obligations of various functional departments, provide a channel for information transmission to achieve barrier-free communication among different functional levels, implement joint and several liability, for example, when a person shows unsafe behavior, and others in the same teams and groups are punished for failing to remind him, so the individual’s unsafe behavior is seen as the whole of unsafe behavior, to promote communication and mutual supervision.

### 5.5. Limitations and Future Research Directions

The findings suggest that potential human risk factors have a significant impact on human behavior, which have both theoretical and practical implications. However, the research reported in this paper does have limitations that should be acknowledged. This paper proposed only general concepts of knowledge, information and communication, and did not describe them specifically. It discussed only the negative impact of knowledge, information and communication on human behavior and didn’t explain its positive impact. For example, how will someone with plenty of professional knowledge affect human behavior? Moreover, this study qualitatively, rather than quantitatively, analyzes the effect of knowledge, information and communication. In addition, we have not yet done a specific intervention study. Based on the theoretical and practical implications of this study, we may develop detailed behavioral interventions and apply them to one or more coal mining companies. Over time, comparing the level of accidents before and after the implementation of the intervention may generate interesting findings and validate our research results in this paper more robustly.

## 6. Conclusions

Potential human risk factors occupy an important position on analyzing human behavior. Potential human risk factors influencing unsafe behavior must be tapped in the study of accidents, so as to fundamentally realize risk control. To the authors’ knowledge, this paper is one of the first studies aiming to discover those potential human risk factors which significantly impact corporation personnel safety behavior, the interactive mechanisms of these factors, and the impact of different functional personnel in the interactive mechanisms. Knowledge, information and communication are potential human risk factors, which evolve through risk latency stage, risk accumulation stage, risk explosion stage and risk residue stage, and different functional personnel make different contributions to their evolution. Major contributions of this paper are as follows:

- The potential human risk factors influencing human behavior were clarified, including knowledge, information and communication. This model proposed in this paper disclosed the interactive mechanism of potential human risk factors.
- Safety is affected by all functional personnel in corporation. Senior managers’ performance is the beginning of the potential human risk evolution. Middle managers’ behavior plays a vital catalytic role. The behavior of on-scene leaders and workers translates the potential risk directly into an accident.

Furthermore, specific practical implications of this paper include the following aspects:

- Corporate personnel must have a high level of knowledge so that the risk can be identified as soon as possible and promptly take mitigation measures.
- Corporate personnel who have a high level of knowledge have effective communication, and then accurately judge the risk information before taking action.
- The level of personnel knowledge should be improve through safety education and training. Through strengthen organizational construction and improve the channels of communication, to achieve the full communication between personnel in the different levels. Implement joint and several liabilities to enhance the effectiveness of communication between personnel in the same level and the completeness of information mastered by corporation personnel.

This paper facilitates a more profound understanding in the interactive mechanisms of knowledge, influence and communication. The specific theoretical and practical implications will also lead BBS researchers to pay more attention to potential human risk factors in order to improve worker behavior intervention effects essentially.

## Figures and Tables

**Table 1:** Unsafe behavior statistics.

Classification	Type	Frequency
Workers’ violation operations	Support improperly	272
	Inspect carelessly in safety inspection	109
	Handle roof-fall accidents illegally	63
	Ignore supervision	59
	Illegal blasting	25
	Cross operation	17
	Workers stand in a wrong place	15
	Remove the pillar in a wrong way	10
	Technical disclosure is irregular	8
	Illegal caving	7
	Work in a wrong order	4
	Support in a wrong form	3
	Tunnel in a wrong way	3
	Wall tapping and roof sounding in a wrong way	2
	Bump the support	2
	Escape to a wrong direction	1
Managers’ violation commands	Issue work commands illegally	90
	Failed to timely work supervise	69
	Failed to formulate complete safety measures	51
	Failed to do a full inspection in safety inspection	48
	Don’t organize an evacuation in a clear sign of roof collapse	20
	Equipped with insufficient staff	8
	Outsource the project to others	4
	Keep escape channel closed	2

**Table 2:** Development analysis on the “4-24” major roof accident.

Accident Risk Stage Division	Unsafe Behaviors	Potential Human-Related Reasons	Classification of Potential Risk Factors
Risk latency stage	Relevant workers went into the working site without accepting safety training.	Managers lacked necessary safety knowledge or didn’t have sufficient understanding of the role of safety training. Workers had little grasp of necessary safety knowledge and safety skills.	K
	The naked old roadway was unsupported and workers worked under an empty supporting roof.	Relevant people didn’t understand whether the naked old roadway can be used again, ignoring the danger of operating under an empty-support roof.	I
	The captain left immediately after mining started.	The captain didn’t perform the duty of patrol and inspection. During the pre-accident omens, the captain didn’t promptly guide workers to take effective measures.	K, C
Risk accumulation stage	The support wasn’t repaired in a timely way after its collapse.	The squad leader thought that repairing the support would delay the progress of the work.	K
	The squad leader didn’t check the operating environment carefully after the support collapse.	The squad leader didn’t think normal work was affected by the supports collapsing and didn’t realize that the collapse of the support increased the danger of the working environment.	I
	The squad leader led workers into the naked old roadway to clean coal after the supports collapsed.	On account of the limitations of their own safety knowledge and risk cognition, workers agreed with the violation instruction of the squad leader and executed the instruction.	K, C
Risk explosion stage	After the supports collapsed, operators were working under an empty-supporting roof until the roof accident occurred.	The squad leader and operators ignored the security problems brought by the collapse of the support.	K, C
		The squad leader didn’t realize the increasing hazard of the face and didn’t detect the pre-accident omen phenomena, so he didn’t command the workers to stop working and to leave the workplace.	K, I
		Workers not only didn’t pass the risk information on to the squad leader, but also didn’t give a different opinion about the dangerous operating environment.	I, C
		Due to the limitations of their own safety knowledge, the lack of communication with workers and sufficient information, government supervisors had failed to provide adequate supervision.	I, C, M
Risk residue stage	Cleaned up roof accident scene without following the related regulations.	Enterprise was focused on production, taking risks to handle the accident scene.	K, I
	Resumed coal mine production without meeting production conditions.	Under conditions of undeveloped repair work, the unfinished task of rectification and lack of risk assessment, the enterprise restarted production operations.	K, I, C

**Table 3:** The correlation analysis results of unsafe behaviors produced by different functional levels’ personnel in coal mines.

Impact Phases	Personal of Each Level	Interaction Degree Collection (A)	Pearson Correlation Coefficient (r)	Sig. (2-Tailed)/(*p* Value)
Level one	senior managers→middle managers	A1	0.941 **	0.000
	senior managers→on-scene leaders	A2	0.882 **	0.002
	senior managers→workers	A3	0.961 **	0.000
Level two	middle managers→on-scene leaders	A4	0.967 **	0.000
	middle managers→workers	A5	0.904 **	0.000
Level three	on-scene leaders→workers	A6	0.925 **	0.000
	on-scene leaders→violation commands	A7	0.950 **	0.004
Level four	workers→violation operations	A8	0.901 **	0.000

Note: ** indicate the corresponding *p* value is less than 0.01.
